# Handwriting Analysis in Children and Adolescents with Hemophilia: A Pilot Study

**DOI:** 10.3390/jcm9113663

**Published:** 2020-11-14

**Authors:** Gianluigi Pasta, Maria Elisa Mancuso, Filomena De Felice, Alexander Seuser, Salvatore Annunziata, Flora Peyvandi, Elena Santagostino, Mario Mosconi, Axel Seuser

**Affiliations:** 1 Operative Unit of Orthopedics and Traumatology, Department of Clinical-Surgical Sciences, Diagnostics and Pediatrics, Fondazione IRCCS Policlinico San Matteo, 27100 Pavia, Italy; gianluigipasta@yahoo.it (G.P.); mario.mosconi@unipv.it (M.M.); 2 Fondazione IRCCS Ca’ Granda, Ospedale Maggiore Policlinico, Angelo Bianchi Bonomi Hemophilia and Thrombosis Center, 20122 Milan, Italy; elisamancuso@gmail.com (M.E.M.); milenadefelice@virgilio.it (F.D.F.); flora.peyvandi@unimi.it (F.P.); e_santagostino@hotmail.com (E.S.); 3 Klinik und Poliklinik für Orthopädie und Unfallchirurgie, Universitätklinikum, 53127 Bonn, Germany; alexander_seuser@yahoo.de; 4 Praxis für Prävention, Rehabilitation und Orthopädie, 53225 Bonn, Germany; axel.seuser@icloud.com

**Keywords:** hemophilia, handwriting, upper limb, sEMG, HJHS

## Abstract

Background: Handwriting is a complex task that requires the integrity of different sensorimotor components to be performed successfully. Patients with hemophilia suffer from recurrent joint bleeds that may occur in the elbow, causing elbow dysfunction with handwriting performance impairment. In our study, we described instrumental dysgraphia that is related to functional disturbances. This pilot study aims to evaluate the handwriting performance in a group of patients with hemophilia. Methods: The study was performed at the Angelo Bianchi Bonomi Hemophilia and Thrombosis Center in Milan. Boys with severe and moderate hemophilia A and B regularly followed-up at that Center, with age between 6–19 years, were eligible. Patients were invited to the Center for one multidisciplinary evaluation of the upper limbs that included: Clinical examination, surface electromyography, and handwriting assessment. Results: All patients, but one, completed handwriting assessment. Overall, 14/19 (74%) had abnormal handwriting, which was overt instrumental dysgraphia in six (32%). There was no difference in Hemophilia Joint Health Score (HJHS) between dysgraphic and non-dysgraphic boys, while surface electromyography (sEMG) revealed a prevalence of flexor muscles of the upper limb in dysgraphic as compared with non-dysgraphic boys. Conclusions: The rather high prevalence of instrumental dysgraphia found in this pilot study deserves a further development of this preliminary experience by increasing the number of examined patients and comparing them with a control group, including quality of life and psychological assessment.

## 1. Introduction

Hemophilia is a chronic bleeding disorder that mainly affects the musculoskeletal system. Despite regular prophylaxis, synovitis and joint damage may occur, and elbows are one of the most commonly affected joints since childhood [1]. Due to tremendous improvements in available treatment, persons with hemophilia (PwH) have, in fact, reached a life expectancy very close to that of the general population, at least in high-income countries [2]. Accordingly, it is becoming increasingly practicable for hemophilia patients to live more “normal lives”, to participate in sport activities, have a professional career, and build a family. Hence, physicians should continue to place greater emphasis on educating, and thereby empowering patients to take control of their hemophilia, and thus, improve their well-being and ability to deal with different life events. A common view is that comprehensive approaches to the management of hemophilia are becoming increasingly important. Studies have shown that key factors influencing the quality of life include family, employment, health, education, and leisure time [3]. Although evidence has shown that both psychosocial factors and clinical conditions play a role in the prediction of quality of life for people with hemophilia [4], the clear definition of those psychological determinants or predictors, which are clues to the understanding of individual differences across clinical states and perceived well-being and quality of life, still needs to be established. As an incurable chronic disease, hemophilia implies a psychological burden both for children and parents that may influence functioning in daily activities [5]. Handwriting is an example of an important life skill that, if impaired, can lead to academic challenges or failure and diminished self-esteem [6,7,8,9]. Moreover, handwriting is a complex task that requires the integrity of different sensorimotor components to be performed successfully. Different types of dysgraphia can be described. In our study, we described “instrumental dysgraphia” that is related to functional disturbances [10]. To our knowledge, no information exists on the handwriting of children with hemophilia. The aim of this pilot study was to assess handwriting performance in a group of children and adolescents with hemophilia to evaluate the impact of this chronic disease on such a skill of everyday life. 

## 2. Materials and Methods

### 2.1. Study Design and Patients

This was a case series in which patients were all evaluated only once in the same session by a multidisciplinary team constituted by a graphologist (F.D.F.), hematologist (M.E.M.), orthopedic surgeon (G.P.), sEMG specialist (Al.S.), and physiatrist (Ax.S.).

Patients with severe or moderate hemophilia A or B aged 6–18 followed-up at the Angelo Bianchi Bonomi Hemophilia and Thrombosis Center in Milan were eligible irrespective of treatment regimen and inhibitor status. Patients were recruited by phone call and invited to come to the Hemophilia Centre for the evaluation.

Patients with recent bleeding episodes of the upper limbs (i.e., within 10 days before enrolment) were excluded.

Written informed consent was obtained from parents/caregivers before examination, and the study was performed according to GCP procedures and the Declaration of Helsinki.

### 2.2. Clinical Assessment

Clinical assessment of dominant and non-dominant upper limb included:

- evaluation of the range of motion (ROM) of cervical spine, shoulder, elbow (flexion-extension and prono-supination), wrist and fingers
- evaluation of wrist and shoulder stability
- evaluation of posture
- calculation of the Hemophilia Joint Health Score (HJHS) 2.0 [11] to assess the elbow joints with a score ranging from 0 (normal joint) to 20 (severe arthropathy)
- evaluation of pressure points (see below for definition) at the same anatomical locations
- temperature measurement at the anatomical locations of pressure points at the elbow (see below)

**Figure 1:**
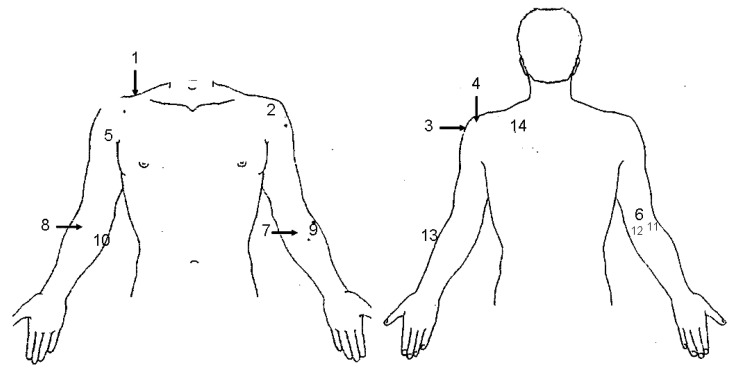
Anatomical sites of detection of pressure points of the upper limb. 1. First rib, 2. Rotator cuff, 3. Subacromial space, 4. Acromioclavicular joint, 5. Biceps tendon, 6. Triceps, 7. Pronator teres, 8. Radial tuberosity, 9. Ligamentum annular radii, 10. Flexor carpi ulnaris, 11. Dorsolateral capsule of the elbow, 12. Dorsomedial capsule of the elbow, 13. Extensor carpi radialis, 14. Levator scapula.

Pressure points were examined to reveal silent symptoms related to the presence of inflammation in capsule, ligaments, and tendons; those points that reacted on pressure were referred to as tender points [12]. Anatomical sites of pressure points are shown in Figure 1.

### 2.3. Temperature Evaluation

The temperature was registered by using a surface thermometer (Tempgun PE1, Pro Exotics, Littleton, CO, USA) [13] at six anatomical sites of pressure points for the elbow (number 6 and 9–13 in Figure 1). All participants had an adaption time to the room for at least 20 min with bare arms. Temperature measurement was taken first before all other measurements. The difference in temperature between dominant and non-dominant limb was calculated and considered significant if ≥0.5 Celsius degrees [14].

### 2.4. Handwriting Analysis

To assess handwriting, participants took down dictation at least 4–5 lines of a fixed cursive text in a sitting position comfortable enough to avoid extrinsic factors that could affect performance (i.e., chair/desk height, type of pen, and paper and its placement on the desk). For the boys, Italian was their first written language.

To describe “instrumental dysgraphia” that is related to functional disturbances, handwriting samples were analyzed using De Ajuriaguerra E and D Scales [15] [Table S1: De Ajuriaguerra E scale; Table S2: De Ajuriaguerra D scale]. The E Scale includes 30 items distinguished into two categories: Form (referred to as “F” items; *n* = 14) and Motor skill (referred to as “M” items; *n* = 16). The first category explores handwriting development according to age and cognitive/emotional status, while the second category is related to motor control and biomechanical issues. For each individual handwriting analysis was based on: (1) The sum of F items, (2) the sum of M items, (3) the sum of the aforementioned sums, and (4) the ratio of F/M sums. D Scale was applied to those cases were the E Scale revealed poor handwriting, according to the age and the scores obtained were used to define the presence of dysgraphia as follows: <10 “no dysgraphia”, 10–14 “probable dysgraphia”, 14,5–19 “should be considered dysgraphic” and >19 “dysgraphia”. Handwriting assessment and functional disturbances diagnosis were performed by a certified specialist on handwriting analysis, as established by Italian law. 

### 2.5. sEMG Measurements

To evaluate muscle function, a 4-channel kinetic sEMG system (Myotrace 400, Noraxon, Scottsdale, AZ, USA) was used. All signals were acquired at a sampling frequency of 1000 Hz [16].

Patients were asked not to train or exercise at least three days before examination in order not to exhaust muscles. The following muscles were assessed: Biceps, triceps, flexor carpi ulnaris, and extensor carpi radialis.

Electrodes (Noraxon HEX Dual Electrodes Product # 272S (spacing—2.0 cm) QTY:8 Disposable, self-adhesive Ag/AgCL snap electrodes) were placed as follows [17]:

- Biceps: Two electrodes (2 cm apart) placed parallel to the muscle fibers in the center of the muscle in correspondence to the maximum circumference.
- Triceps: Two electrodes (2 cm apart) placed parallel to the muscle fibers, 2 cm medial from the midline of the arm, approximately 50% of the distance between the acromion and the olecranon.
- Flexor carpi ulnaris: Two electrodes (2 cm apart) placed parallel to the muscle fibers over the muscle mass, approximately 50% of the distance between the elbow and the wrist.
- Extensor carpi radialis: Two electrodes (2 cm apart) placed parallel to the muscle fibers over the muscle mass 5 cm distal from the lateral epicondyle of the elbow.

The measurements were performed in three different functional conditions: (i) Resting tonus of the muscle; (ii) maximum isometric contraction (neutral position against resistance); (iii) isotonic repetitions (muscle contraction repeated three times), which corresponded to the typical muscle function, e.g., flexion/extension of the elbow and flexion/extension of the wrist.

Resting tonus was considered normal when <2.5 microVolts.

Muscles were assessed during isometric contraction on both sides, and results compared to evaluate side differences. A side difference was considered acceptable when <15%.

During isotonic motion, the sEMG curve displays three main portions: An ascending phase corresponding to muscle contraction (referred to as concentric phase or concentric contraction), a change of direction of the curve (referred to as intermediate phase), and a descending phase corresponding to muscle decontraction (referred to as excentric phase). Concentric contraction measured on both sides was compared to evaluate side differences. A side difference was considered acceptable when <15%.

A correlation coefficient that measures accuracy during isotonic repetitions was calculated, being 0.90–1.00 the values associated with the most accurate outcome [18]. 

### 2.6. Statistical Analysis

The distribution of all continuous variables was tested by Kolmogorov-Smirnov test, and parametric or non-parametric tests for analysis were applied accordingly. Continuous variables were expressed as median values and interquartile ranges (IQR) and were compared by the t-Student or Mann-Whitney U tests. Categorical variables were expressed as frequencies and percent values and compared by chi-square or Fisher’s exact test. All *p* values reported are two-sided, and a value <0.05 was considered statistically significant. All analyses were performed by using IBM SPSS Statistics software (release 25.0, IBM Corp., Armonk, NY, USA).

## 3. Results

Overall, 20 patients accepted to be enrolled in the study and came to the Hemophilia Center on two consecutive days (10 patients per each day).

The main characteristics of the patients are shown in Table 1. Body mass index (BMI) was normal in all included individuals.

Two patients, treated on-demand, included one moderate hemophilia A and one moderate hemophilia B patient. Among those treated on prophylaxis: Eleven patients had no inhibitor history, four had been successfully tolerized at least two years before the study, two had achieved partial response after immune tolerance induction and were on daily high-dose FVIII prophylaxis, and one had current high-titer inhibitors and was on activated prothrombin complex concentrate (aPCC) prophylaxis. 

Nine patients (45%) had at least one joint bleed in the elbow in their medical history, and six of them had a joint bleed in the elbow in the year before the study.

### 3.1. Clinical Assessment

#### 3.1.1. ROM

Only one patient had a reduced ROM of the cervical spine.

Five patients had a reduced ROM of external (*n* = 5) or internal (*n* = 1) rotation (from −10° to −50°) of the shoulder. Only one patient had a reduced ROM of abduction and elevation of the non-dominant shoulder. 

Six patients had a reduced ROM of the dominant elbow: All of them had a reduced arc of movement in flexion-extension (from −5° to −20°), and only two had reduced pronation (−5° and −10°, respectively). In two patients, the ROM of the non-dominant elbow was also impaired.

Two patients had a reduced ROM of the dominant wrist (−5° and −10°, respectively). 

#### 3.1.2. HJHS 2.0

The median HJHS of the dominant elbow was 0 (range: 0–3), and seven patients (35%) had an HJHS >0.

Only two patients had a positive score for pain at HJHS evaluation at the dominant elbow. 

#### 3.1.3. Tender Points

All patients, but two, had tender points in the dominant upper limb (median: 4, range: 1–12). Among those 18, the most prevalent tender points were found at the following anatomical sites: Levator scapula (15, 83%), Ligamentum annular radii (11, 61%), first Rib (9, 50%), Extensor carpi radialis (8, 44%) and Biceps tendon (7, 39%).

#### 3.1.4. Temperature Measurement

A temperature difference >0.5 °C being higher at the dominant upper limb as compared with the non-dominant was found at the following sites for pressure points: Extensor carpi radialis (7, 35%), ligamentum annular radii (6, 30%), dorsolateral capsule (6, 30%), dorsomedial capsule (8, 40%), triceps (7, 35%) and flexor carpi ulnaris (10, 50%). The positive predictive value of the presence of tender points at sites with a temperature difference >0.5 °C ranged between 0.10 (flexor carpi ulnaris) to 0.67 (ligamentum annular radii and dorsolateral capsule), while the negative predictive value ranged between 0.50 (ligamentum annular radii) and 0.93 (dorsolateral capsule). 

Temperature resulted invariably higher in the absence of tender points in four children who had arteriovenous fistula surgically created at the elbow crease as alternative venous access to inject regularly as per prophylactic treatment [19].

#### 3.1.5. Handwriting Analysis

**Figure 2:**
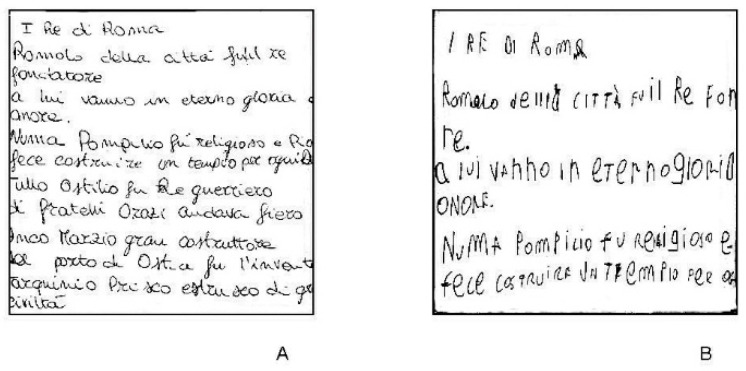
Handwriting samples: (**A**) Non-dysgraphic patient; (**B**) dysgraphic patient.

All patients, but one, completed handwriting assessment. According to the scales used, four patients (21%) had no dysgraphia, and six (32%) had overt dysgraphia. Eight patients (42%) should be considered dysgraphic (scores between 14.5 and 19) and only one (5%) had probable dysgraphia. Hence, overall, 14 patients (74%) had features of dysgraphic handwriting. Figure 2 shows one handwriting sample from a non-dysgraphic (panel A) and one from a dysgraphic patient (panel B). 

Median HJHS scores were similar for both elbows comparing dysgraphic and non-dysgraphic patients. 

#### 3.1.6. sEMG Measurements

Overall, the median resting tonus measured for biceps, triceps, flexor carpi ulnaris, and extensor carpi radialis was normal (<2.5 microVolts) for both dominant and non-dominant limb in all patients. Although higher absolute values were invariably found for all muscles and in both limbs in dysgraphic patients, the difference in comparison with non-dysgraphic patients did not reach statistical significance (see supplementary materials). 

Isometric contraction microvoltage of triceps, flexor carpi ulnaris, and extensor carpi radialis was similar in dysgraphic and non-dysgraphic patients, while that of biceps was significantly higher for both limbs in dysgraphic patients (Table S3: Results of sEMG measurements performed in 14 dysgraphic and five non-dysgraphic patients enrolled in the study). The median side difference in isometric contraction was >15% for all muscles, both in dysgraphic and non-dysgraphic patients. 

The median concentric contraction of all examined muscles was similar by comparing dysgraphic and non-dysgraphic patients (see supplementary materials). The median side difference in concentric contraction was >15% for all muscles, both in dysgraphic and non-dysgraphic patients. 

The median correlation coefficient measured for all examined muscles was between 0.7 and 0.9 for both dysgraphic and non-dysgraphic patients (see supplementary materials). 

**Figure 3:**
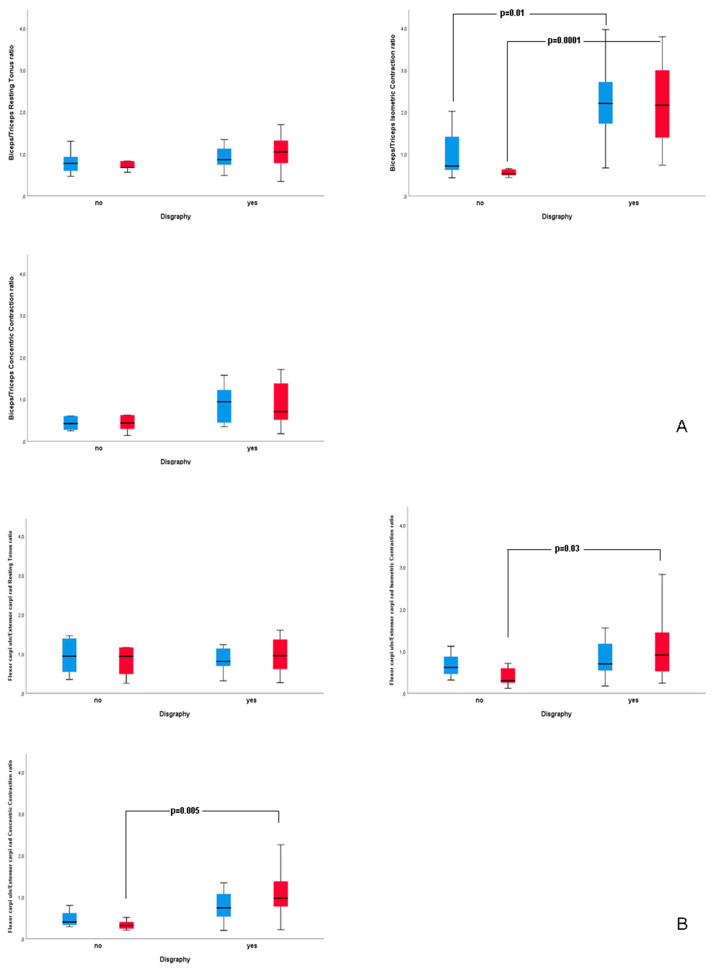
Flexors/Extensors (Biceps/Triceps, panel **A** and Flexor carpi ulnaris/Extensor carpi radialis, panel **B**) ratios for main sEMG parameters measured for dominant (red bars) and non-dominant (blue bars) upper limb in dysgraphic and non-dysgraphic patients enrolled in the study.

Figure 3 shows the flexors/extensors (i.e., biceps/triceps and flexor carpi ulnaris/extensor carpi radialis) ratios for the main sEMG parameters calculated for dominant and non-dominant limb and compared between dysgraphic and non-dysgraphic patients. The median biceps/triceps ratios for isometric contraction were significantly higher in dysgraphic vs. non-dysgraphic patients for both dominant (2.2 vs. 0.5, IQR: 1.3–3.0 vs. 0.4–0.6; *p* = 0.0001) and non-dominant (2.2 vs. 0.7, IQR: 1.7–2.8 vs. 0.5–1.7; *p* = 0.01) limb revealing a prevalence of flexors over extensors of the arm in dysgraphic patients. On the contrary, a prevalence of extensor carpi radialis over flexor carpi ulnaris in the dominant limb was found in non-dysgraphic patients as compared with dysgraphic patients (median flexor carpi ulnaris/extensor carpi radialis ratio for isometric contraction of dominant limb 0.3 vs. 0.9, R: 0.2–0.7 vs. 0.5–1.5, respectively, *p* = 0.03; median flexor carpi ulnaris/extensor carpi radialis ratio for concentric contraction of dominant limb 0.3 vs. 1.0, IQR: 0.2–0.5 vs. 0.7–1.4, respectively, *p* = 0.005).

## 4. Discussion

In this pilot study, focused on handwriting analysis in a group of children and adolescents with hemophilia, we found a rate of dysgraphia much higher than that described in the general population. Estimates of the incidence of poor performance in typically developing children range from 10 to 30% [20]. In the study group, we observed dysgraphic features in up to 74% of cases. This could be due to the small sample size of our study and also to different methods used to evaluate handwriting in different studies; however, it represents a signal of a possible problem present in children and boys with hemophilia. 

Handwriting is an example of an important life skill that, if impaired, can lead to academic challenges or failure and diminished self-esteem [6,7,8,9]. Although most children are now exposed to computers at an early age, handwriting is still used in classrooms and everyday life. Research has shown that children spend 31–60% of their school days performing fine motor tasks, and 85% of this time is devoted to paper-pencil tasks, such as handwriting [20]. This has not changed much in the last decade, despite the increasing integration of computers into the school environment. Moreover, handwriting is a complex task that requires the integrity of different sensorimotor components to be performed successfully. Kinesthesia, motor planning, in-hand manipulation, and visual-motor skills are associated with good handwriting legibility in typically developing children. Cognitive and psychosocial components are also known to influence handwriting performance [21]. 

Normal handwriting depends on neural control. It demands a constant accurate afferent input of mechanoreceptors of joints and associated structures, adequate interpretation of those inputs, and a decent motoric answer. Moreover, to be adequately performed, it needs good motor control with intermuscular coordination and load transfer on articular structures in the right areas. Hence, it needs an optimal force closure based on specific characteristics of each single involved muscle as endurance, strength, flexibility, and tensegrity. Besides a good muscle function, the integrity of osteo-ligamentous structures is also important. In fact, the more joint function is disturbed by ligament overload, inflammation, and/or synovial hypertrophy, the higher is the likelihood of functional imbalance. 

The original idea of the present study was that handwriting could be a model for the function of upper extremities as gait is for lower extremities [22,23]. To our knowledge, this is the first study that assessed handwriting and muscle function of the upper limb with kinetic sEMG in patients with hemophilia.

To evaluate the different parts of the integrated model of joint function [24,25], we used HJHS, tender points, and temperature measurement for structural evaluation, and there were no major findings in our patient group. We found some evidence of silent low-grade inflammation in ligaments and capsules around the elbow joint. 

Kinetic superficial EMG was used for the evaluation of force closure. Isometric contraction of biceps was significantly higher in boys with instrumental dysgraphia. This indicates a starting contraction and the necessity to stretch. It is well-known that elbows develop flexion contraction in the course of hemarthropathy; this might be the first step of the functional impairment that precedes structural involvement, and it is likely that this dysfunction might influence the task of handwriting. Also, the finding of a median side difference in the isometric and isotonic contraction of more than 15% in all our patients might suggest a very early functional involvement on both elbows.

Looking at muscle interactions on the level of motor control, we could show a prevalence of flexors over extensors (i.e., biceps and triceps) of the upper arm in boys with dysgraphia and of extensors over flexors (i.e., extensor and flexor carpi) for the wrist muscles in non-dysgraphic boys. This might indicate an initial deterioration of motor control that starts pushing the muscular control balance to the flexor side. The described biomechanical changes can explain, at least in part, the high incidence of different degrees of severity in dysgraphia in our patients. 

The lack of correlation with HJHS suggests that there are aspects of muscle function impairment that are not detected by such clinical scores; due to the small sample size of the current study, these results need to be confirmed in a larger cohort of patients. 

Nonetheless, function impairment usually precedes structural damage, hence it is importance to include more sensitive measurement tools for a more accurate musculoskeletal examination of patients with hemophilia. Handwriting analysis and sEMG are good candidates because they are relatively cheap and easy to perform as compared with motion and/or gait analysis. 

Moreover, as muscle dysfunction can be rehabilitated, the detection of early changes may foster the implementation of individualized training programs, which could include corrective interventions for dysgraphia as well. 

Besides the novelty of our study, we recognize some limitations: The limited number of patients included, the lack of a control group of non-hemophilic age-matched males from the same geographic area, the lack of imaging examination of elbows and of quality of life/psychological assessment.

## 5. Conclusions

The goal of our multidisciplinary study group is to further develop this preliminary experience by increasing the number of examined patients and comparing them with a control group. Moreover, elbow joint assessment through ultrasound and/or MRI, quality of life assessment through validated questionnaires, and psychological assessment will be included to confirm the high rate of dysgraphia observed in this pilot study and to investigate the role of all the possible determinants of dysgraphia in this patient population.

## Figures and Tables

**Table 1:** Main characteristics of the 20 patients enrolled in the study.

	Patients (*n* = 20)
Median age at enrolment, yrs (IQR)	12 (9–16)
Hemophilia A (%)	19 (95)
Severe hemophilia (%)	18 (90)
Patients on regular prophylaxis ^†^ (%)	18 (90)
Patients treated ≥3 times/week (%)	16 (80)
Right-handed (%)	18 (90)

^†^ Here regular prophylaxis is FVIII/FIX infusions given at least once weekly.

## Supplementary Materials

The following are available online at https://www.mdpi.com/2077-0383/9/11/3663/s1, Table S1: De Ajuriaguerra E Scale, Table S2: De Ajuriaguerra D Scale, Table S3: Results of sEMG measurements performed in 14 dysgraphic and 5 non-dysgraphic patients enrolled in the study.

## Abbreviations

aPCC	activated prothrombin complex concentrate
HJHS	Hemophilia Joint Health Score
PwH	persons with hemophilia
ROM	range of motion
sEMG	surface electromyography
