# Supplementary material for: Handwriting Analysis in Children and Adolescents with Hemophilia: A Pilot Study
Source: J Clin Med. 2020 Nov 14;9(11):3663. doi: 10.3390/jcm9113663 (PMC7697872; doi:10.3390/jcm9113663)
Supplement: Supplementary file 1 [file jcm-09-03663-s001.pdf]

Table S1. De Ajuriaguerra E Scale.

	Item	*Points*	*Coeff. Pond.*	*TOT*
	**EF**			
F1	Superficial writing, immature		2	
F2	Dodue writing (distended, chubby)		1	
F3	Without movement		2	
F4	High caliber		2	
F5	Calligraphic “m” and “n”		2	
F6	Calligraphic “t” cuts		2	
F7	Calligraphic “p” letter		1	
F8	“a” letter in two traits		3	
F9	“d”, “g”, “q” letters in two traits		2	
F10	Capital letters drawn in a clumsy way		3	
F11	Sutures		3	
F12	Collages		1	
F13	Irregular space between lines		3	
F14	Undifferentiated zones		2	
		**EF TOT**		
	**EM**			
M15	Descending trait interrupted or picked up		3	
M16	Letters with touch-up or corrections		3	
M17	Tethered array with smears		3	
M18	Rod deviations of “d”, “t”, “p”, q”		1	
M19	Dented loops		3	
M20	Awkwardness of loop “g”, “f” etc		2	
M21	Tremor		3	
M22	Shaking layout		2	
M23	Snap writing		2	
M24	Crowded letters		2	
M25	Broken line		2	
M26	Sinuous line		1	
M27	Descending line		1	
M28	Dancing words on the line, snapping		2	
M29	Dimension irregularity		3	
M30	Direction irregularity		1	
		**EM TOT**		

Table S2. De Ajuriaguerra D Scale.

		Score	*Coeff. Pond.*	*Score tot.*
	**Wrong use of space**			
D1	Dirty and tethered array		1	
D2	Broken line		1	
D3	Rolling line		2	
D4	Descending line		1	
D5	Packed between words		2	
D6	Irregular space between words		1	
D7	Absence of margins		1	
	**Awkwardness**			
D8	Poor trait		2	
D9	Retouched or corrected letters without reason		2	
D10	Loops filled with ink		1	
D11	Twisting in the median zone		1	
D12	Sharp arcades		1	
D13	Sutures or weldings		2	
D14	Collages		1	
D15	Crowded letters		3	
D16	Snap writing		2	
D17	Slender finals related to awkwardness		2	
D18	Dimension irregularity		2	
D19	Bad differentiated zones		1	
D20	Atrophied letters		2	
	**Mistakes in form and proportion**			
D21	Structured or tenuous letters		2	
D22	Poor shapes		1	
D23	Too little or excessive dimension		2	
D24	Bad ratio between zones		2	
D25	Too distended or packed writing		1	
		**TOT score**		

Table S3. Results of sEMG measurements performed in 14 dysgraphic and 5 non-dysgraphic patients enrolled in the study.

Muscle	Parameter	Side	Dysgraphic patients (n=14) *Median value (IQR)*	Non-dysgraphic patients (n=5) *Median value (IQR)*	p value
**Biceps**	Resting tonus, μV	D	1.4 (1.0-1.9)	0.9 (0.7-1.5)	ns
		ND	1.3 (1.1-1.6)	1.0 (0.6-1.4)	ns
	Mean isometric contraction, μV	D	164 (74-222)	38 (20-81)	0.01
		ND	116 (74-189)	29 (20-124)	0.03
	Mean concentric contraction, μV	D	17 (11-37)	10 (6-39)	ns
		ND	18 (10-41)	8 (6-35)	ns
	Correlation coefficient	D	0.8 (0.6-0.9)	0.7 (0.6-0.9)	ns
		ND	0.7 (0.4-0.8)	0.5 (0.3-0.8)	ns
**Triceps**	Resting tonus, μV	D	1.3 (1.0-1.8)	1.0 (0.9-1.8)	ns
		ND	1.5 (1.2-1.6)	1.2 (1.0-1.7)	ns
	Mean isometric contraction, μV	D	66 (54-79)	72 (44-127)	ns
		ND	62 (37-82)	48 (22-97)	ns
	Mean concentric contraction, μV	D	27 (24-36)	33 (23-46)	ns
		ND	27 (19-36)	28 (17-37)	ns
	Correlation coefficient	D	0.8 (0.7-0.9)	0.8 (0.5-0.9)	ns
		ND	0.8 (0.6-0.9)	0.8 (0.7-0.9)	ns
**Flexor carpi ulnaris**	Resting tonus, μV	D	1.5 (1.2-2.3)	1.4 (1.0-1.7)	ns
		ND	1.6 (1.1-2.0)	1.3 (0.9-1.6)	ns
	Mean isometric contraction, μV	D	60 (37-96)	18 (5-83)	ns
		ND	53 (40-97)	52 (9-82)	ns
	Mean concentric contraction, μV	D	30 (26-60)	11 (5-36)	ns
		ND	28 (17-54)	22 (6-56)	ns
	Correlation coefficient	D	0.9 (0.7-0.9)	0.7 (0.5-0.9)	ns
		ND	0.8 (0.7-0.9)	0.9 (0.7-0.9)	ns
**Extensor carpi radialis**	Resting tonus, μV	D	1.7 (1.2-3.3)	1.4 (1.0-2.7)	ns
		ND	1.6 (1.3-2.7)	1.4 (1.0-2.5)	ns
	Mean isometric contraction, μV	D	69 (49-107)	61 (37-129)	ns
		ND	75 (59-101)	19 (12-94)	ns
	Mean concentric contraction, μV	D	56 (26-66)	55 (17-83)	ns
		ND	45 (28-66)	20 (11-64)	ns
	Correlation coefficient	D	0.7 (0.7-0.9)	0.8 (0.7-0.9)	ns
		ND	0.8 (0.7-0.9)	0.8 (0.7-0.9)	ns

D= dominant side, ND= non dominant side.
